# Current Status of Septic Cardiomyopathy: Basic Science and Clinical Progress

**DOI:** 10.3389/fphar.2020.00210

**Published:** 2020-03-03

**Authors:** Huan Lin, Wenting Wang, Madeline Lee, Qinghe Meng, Hongsheng Ren

**Affiliations:** ^1^Department of Intensive Care Unit, Shandong Provincial Hospital Affiliated to Shandong University, Jinan, China; ^2^Dartmouth College, Hanover, NH, United States; ^3^Department of Surgery, SUNY Upstate Medical University, Syracuse, NY, United States

**Keywords:** sepsis, heart failure, septic cardiomyopathy, pathogenesis, treatment, diagnosis, left ventricular ejection fraction

## Abstract

Septic cardiomyopathy (SCM) is a complication that is sepsis-associated cardiovascular failure. In the last few decades, there is progress in diagnosis and treatment despite the lack of consistent diagnostic criteria. According to current studies, several hypotheses about pathogenic mechanisms have been revealed to elucidate the pathophysiological characteristics of SCM. The objective of this manuscript is to review literature from the past 5 years to provide an overview of current knowledge on pathogenesis, diagnosis and treatment in SCM.

## Introduction

Sepsis is defined as a life-threatening organ dysfunction due to a dysregulated immune response to an infection (Singer et al., 2016) and has become one of the top ten leading causes of death in developed and developing countries (Hawiger, 2018), with a mortality rate as high as 30% (Morin et al., 2015). Septic shock is defined as hypotension with lactic acid >2 mmol/L after adequate fluid resuscitation (Singer et al., 2016) and its death rate can be as high as 50% (Lu et al., 2019). First described over 40 years ago, septic cardiomyopathy (SCM) is an acute cardiac disorder caused by sepsis (Beesley et al., 2018), which is reversible and can be restored at an early stage of sepsis (Lu et al., 2019). The reported incidence of SCM in patients with sepsis or septic shock is inconsistent, ranging from 13.8 to 40% due to lack of large-scale studies and uniform diagnostic criteria (Li et al., 2019; Lu et al., 2019). In patients with sepsis combined with SCM, the mortality is increased 2–3 times, up to 70–90% (Ehrman et al., 2018; Fan and Zhang, 2018; Li et al., 2019). Epidemiological studies have shown that male, younger age, higher lactate level and previous history of heart failure/coronary heart disease as well as lactic acid level (>4.0 mmol/L) when admitted to ICU are associated with SCM (Sato et al., 2016; Li et al., 2019). Other evidences have shown that acute physiology and chronic health evaluation system II score (Caser et al., 2014) and the application of catecholamine were positively correlated with occurrence of SCM (Wang and Li, 2015).

The myocardial depressant factors (MDFs) have been proposed since the 1950s (Lefer, 1982; Fan and Zhang, 2018), referring to active substances that suppress the action of the heart. Currently, recognized MDFs include cytokines, the complement system, nitric oxide (NO), lipopolysaccharides (LPS) etc. Cytokines include tumor necrosis factor-α (TNF-α), interleukin-1β (IL-1β), interleukin-6 (IL-6), and high mobility protein box 1 (HMGB1) (Beesley et al., 2018; Lu et al., 2019). Moreover, the activations of Toll like receptors (TLRs) and nuclear factor kappa B (NF-κB) contribute to pathogenesis of SCM (Martin et al., 2019). In general, SCM is defined as the decrease in left ventricular ejection fraction (LVEF) and ventricular dilatation during sepsis (Beesley et al., 2018). Clinically, in addition to proper management of infection and sepsis control, maintenance of hemodynamic stability is the first step for patients with SCM. The currently recommended treatment strategy is symptomatic, and there is no specific treatment in septic patients with SCM.

Some progress has been made in understanding the pathogenesis of SCM. In recent years, there are excellent review articles regarding the pathophysiology of cardiomyopathy, present review focuses on the current the evidence on pathogenic mechanism of SCM and the potential of diagnoses and treatments for SCM.

## Pathogenesis

Presently, the pathogenesis of SCM is still in its exploration stage. The role of inflammatory cytokines in the pathogenesis of sepsis was identified. But all antibodies based on specific pro-inflammatory cytokines as targets have failed in clinical trials (Kumar, 2019). This indicates that some others factors are involved in the pathogenesis of SCM like mitochondrial dysfunction and exosomes in cardiac myocytes, suggesting that SCM is caused by many contributing factors. We will highlight recent discoveries of pathogenic mechanisms that are associated with SCM.

### The Complement System

Sepsis can lead to the activation of the complement system, resulting in increasing the amount of complement component 5 (C5a). C5a reacts with its receptor, leading to cytokine storm, lymphocyte apoptosis, neutrophilic innate immune function loss, cardiomyopathy, disseminated intravascular coagulation, etc. Meanwhile, C5a also affects intracellular calcium homeostasis (Fattahi et al., 2018a; Ward and Fattahi, 2019). SCM is associated with decreased levels of three key enzymes (serca2, NCX, and Na+/K+-atpase) in cardiomyocytes, which are complement receptor-dependent (Fattahi et al., 2018a). Some studies have suggested that histones may be a target for reducing cardiac dysfunction in sepsis. Interestingly, the investigation of mechanism has found that extracellular histones appearing in sepsis plasma require C5a receptors (Kalbitz et al., 2015). A study evaluated the cardiac function and contractility of cardiomyocytes in rats with cecal ligation and puncture (CLP). The left ventricular pressure decreased significantly. These defects were prevented in CLP rats by blocking antibody against C5a. After the addition of recombinant rat C5a, both sham and CLP myocardial cells showed significant systolic dysfunction. These data indicates that CLP induces the generation of C5a receptor by cardiomyocytes, and the production of C5a leads to the interaction of C5a-C5a receptor, leading to the dysfunction of cardiomyocytes, then resulting in the impairment of cardiac function, suggesting interventions directly targeted at C5a interception or C5a receptor blockade may be a new and promising treatment for patients with SCM (Niederbichler et al., 2006). A study from Keshari et al. has shown that inhibition of C5 protects organ failure and reduces mortality in baboon model of sepsis via decreasing plasma LPS concentration and inhibiting the production of inflammatory cytokines. In addition, C5 inhibitor attenuated sepsis-increased soluble uPAR, thrombomodulin and angiopoietin-2 in plasma, suggesting that C5 inhibition may also protect against endothelial cell dysfunction (Keshari et al., 2017). Another study using Soliris (Eculizumab), an FDA-approved C5 inhibitor, for the treatment of paroxysmal nocturnal hemoglobinuria (PNH) has shown that Eculizumab application in a septic child rescued the sepsis-induced multiorgan failure, including cardiac dysfunction (Galic et al., 2019). All the above studies indicate that C5 is closely related to the occurrence of SCM, and suggesting that a C5 inhibitor may be a promising treatment for patients with SCM.

### Mitochondrial Dysfunction in Cardiac Myocytes

Cardiomyocytes have a high mitochondrial density, which allows them to produce adenosine triphosphate (ATP) quickly. ATP provides the energy for both energy-consuming endergonic reactions and energy-releasing exergonic reactions. Mitochondrial dysfunction can seriously affect heart function in sepsis. Mitochondrial damage occurs in SCM, mainly manifested as morphological and functional changes, including interrupted oxidative phosphorylation, impaired mitochondrial respiration rate, free radical production of mitochondria, decreased mitochondrial membrane potential, attenuated autophagy, and apoptosis (Tan Y. et al., 2019). Takasu et al. (2013) conducted autopsies on 17 patients who died of sepsis and have found that six of them had mitochondrial micro-structural damage including mitochondrial swelling, ridge loss, as well as rupture of inner and outer membrane. They have demonstrated that the integrity of the mitochondrial micro-structure is necessary to ensure that mitochondria produces enough energy. When the micro-structure is damaged, mitochondria becomes dysfunctional. Durand et al. (2017) have discussed mitochondrial oxidative phosphorylation disorder in SCM, and pointed out that reactive oxygen species (ROS) and other substances, such as cytochrome C produced in mitochondria, were considered as a signal related to apoptosis. BAP31, a B cell receptor-related protein, may affect mitochondrial homeostasis and endoplasmic reticulum function. The transcription of BAP31 was inhibited in LPS-treated cardiomyocytes. While melatonin could up-regulate the expression of BAP31, this effect depends on the MAPK-ERK pathway. Inhibition of the ERK pathway and/or inhibition of BAP31 could diminish the beneficial effects of melatonin on mitochondrial function and endoplasmic reticulum homeostasis under LPS stress, suggesting that ERK-BAP31 pathway can be a regulator of mitochondrial function and endoplasmic reticulum homeostasis (Zhang et al., 2019). Yes- related protein (Yap), a transcriptional activator in the Hippo signaling, plays an important role in mitochondrial function, especially mitochondrial fission function, which is associated with a variety of cardiovascular diseases. In a mouse model of SCM, LPS down-regulated the expression of Yap, while Yap overexpression can maintain cardiac function and reduce myocardial cell death via regulating mitochondrial fission (Yu et al., 2019). Chen et al. (2019a) found that the myocardial cells in septic mice were significantly overexpression of long non-coding RNA (LncRNA) SOX2 overlapping transcript (SOX2OT), suggesting that SOX2OT contributed to mitochondrial dysfunction in SCM. Receptor-interacting protein kinase3 (Ripk3) may regulate signaling pathways that are related to mitochondrial injury, endoplasmic reticulum stress, and cell scaffold balance. Zhong et al. (2019) found that Ripk3 expression was increased in LPS-infected cardiomyocytes. Mitochondrial autophagy plays an integral role in cardiac dysfunction. A study has shown that Beclin-1, an autophagy protein, modulates inflammation and improves cardiac function in the LPS-induced animal sepsis (Sun et al., 2018).

In summary, the findings from experimental animal models have shown that changes in mitochondrial morphology and function are involved in the pathogenesis of SCM. Understanding molecular mechanisms of mitochondrial injury may provide evidence for developing new therapeutic targets for SCM.

### Toll-Like Receptors in Cardiomyocytes

Toll-like receptors (TLRs), a trans-membrane glycoprotein on the surface of the cell membrane, is an important part of the immune system that can identify different pathogen associated molecular patterns (PAMPs). The stimulation of TLRs by PAMPs causes the nuclear translocation of nuclear factor kappa B (NF-κB), and then leads to the expression of inflammatory mediators, such as TNF-α and interleukins (ILs) (Dalton and Shahul, 2018). Signaling regulated by TLRs is classified MyD88-dependent and MyD88-independent pathways. TLRs form homodimers, and one or more adaptor proteins such as MyD88, MAL/TIRAP, TRIF or TRAM, which are then recruited into the cytoplasm after the binding of TLRs to their respective ligands. MyD88 dependent pathway is utilized by most TLRs except TLR3 (Feng and Chao, 2011). In MyD88-dependent pathway, MyD88 binds IRAK4, IRAK1, and/or IRAK2, and promotes their binding to TRAF6, then leads to the activation of TAK1 by TRAF6. After a series of activations/reactions, this eventually leads to nuclear translocation of NF-κB, which activates the expression of various inflammatory genes (called gene storms), causing host dysfunction and multiple organ dysfunction (Hawiger, 2018). In MyD88 independent pathways, such as Trif-dependent pathways, Trif interacts with TRAF3 to activate TBK1 and IKKi, and then cause the phosphorylation of IRF3. Phosphitylated IRF3 is transferred into the nucleus to activate type I IFN and IFN-induced gene transcription. Myocardial cells express TLR2, TLR3, TLR4, and TLR9 (Vallejo, 2011). A study by Fattahi et al. (2018b) has shown that LVEF was increased and plasma pro-inflammatory cytokines (TNF-α, IL-1, IL-6) were decreased significantly in a mouse septic model with TLR9 and TLR3 deletion, suggesting that TLR9 and TLR3 activation is associated with dysfunction of heart in sepsis. TLR4 can bind to LPS, and then cause the release of a variety of inflammatory factors, finally insults in cardiac dysfunction (Vallejo, 2011). TLR4 regulates oxidative stress in ryanodine receptor 2 (RyR2), leading to increased Ca^2+^ leakage in the sarcoplasmic reticulum (SR) of cardiac myocytes (Yang et al., 2018). Chen et al. (2019b) analyzed gene expression in septic patients compared with control, showing that TNF-α, JAK and transcriptional activation (STAT) signaling pathways were up-regulated. Cirulis et al. (2019) provided the evidence for the role of interferon signaling in SCM using a human study. The linkage between activations of TLRs/its downstream signals and SCM has been established from current investigations. Inhibition of TLR4 has shown protective effect on SCM in experimental animal models (Fenhammar et al., 2014; Yang et al., 2018). Based on those findings, targeting TLRs to develop new therapeutic approaches is promising.

### Nitric Oxide and Nitric Oxide Synthase in Cardiac Myocytes

Nitric oxide is synthesized by the oxidation of L-arginine by nitric oxide synthase (NOS) expressed in cardiac myocytes. NOS can be divided into three subtypes, namely neuronal nitric oxide synthase (nNOS), inducible nitric oxide synthase (iNOS), and endothelial nitric oxide synthase (eNOS) (Martin et al., 2019). The only small amount of NO produced by nNOS and eNOS are noted in physiological state, but NO produced by eNOS plays a protective role in vascular endothelium and vascular function (Mingjie and Zheng, 2018). iNOS are not responsible for producing NO in normal physiological state. However, iNOS will produce a large amount of NO when an inflammatory response occurs (Martin et al., 2019). In humans, neutrophils also express iNOS. Bacterial invasion leads to the activation of TLRs, which causes elevated inflammatory meditators (cytokines, chemokines etc.) that overstimulate neutrophils, then cause the expression of iNOS and subsequently increase the production of NO, finally result in up-regulation of G-protein-coupled receptor kinase 2 (GRK2), down-regulation of CXC chemokine receptor 2 (CXCR2), shedding of L-selectin, decrease in adhesion molecules, and influence of neutrophil chemotaxis (Spiller et al., 2019). A number of experiments have confirmed that the production of NO by iNOS can impair heart function, such as down-regulating adrenaline receptors, decrease sensitivity of myocardium to Ca^2+^ and also damage to mitochondria, etc. (Martin et al., 2019). We have discussed the role of mitochondrial dysfunction in cardiac myocytes in SCM previously. A study has shown that melatonin (an iNOS inhibitor) prevents the destruction of mitochondrial homeostasis after sepsis, restores ATP production and improves the survival rate of sepsis (Cimolai et al., 2015). This evidence supports the hypothesis that mitochondrial homeostasis and increased NO play a role in the pathogenesis of SCM.

### Nicotinic Acetylcholine Receptor α7 Subunit

Alpha 7 nicotinic acetylcholine receptor (α7nAchR) is widely expressed in the cytokine-producing immune cells such as macrophages, dendritic cells and T cells (Souza et al., 2019). α7nAchR is an important element of the cholinergic anti-inflammatory pathway (CAP). Acetylcholine (Ach), a neurotransmitter released by stimulation of vagus nerve, binds to α7nAchR on cell surface and inhibits the degradation of NF-κB inhibitory proteins through a series of intracellular signals, preventing its separation from NF-κB, thereby inhibiting the translocation of NF-κB and reducing the release of pro-inflammatory cytokines (Chao et al., 2015). The evidences from some studies have shown that the expression of α7nAchR in LPS-induced septic mouse model is significantly decreased compared with control group (Kong et al., 2017). At the same time, dexmedetomidine can reduce the expressions of apoptotic proteins, IL-6, IL-1, and TNF-α through α7nAchR activation, thus protecting the myocardium in septic mice. GTS-21, a synthetic selective stimulant of α7nAchR, has been shown to reduce myocardial injury via modulating inflammation (decreases in IL-6, IL-1β, TNF-α and activation of NF-κB P65) and apoptosis in LPS-induced sepsis in mice (Kong et al., 2018). Although there are a few studies on the activation of CAP in this area, the beneficial role of CAP activation in SCM will be emphasized in the future because α7nAchR is an essential mechanism for the CAP which has revealed potent immunomodulatory properties in various diseases including SCM.

### The Effects of Exosomes on Cardiac Function in Sepsis

**FIGURE 1:**
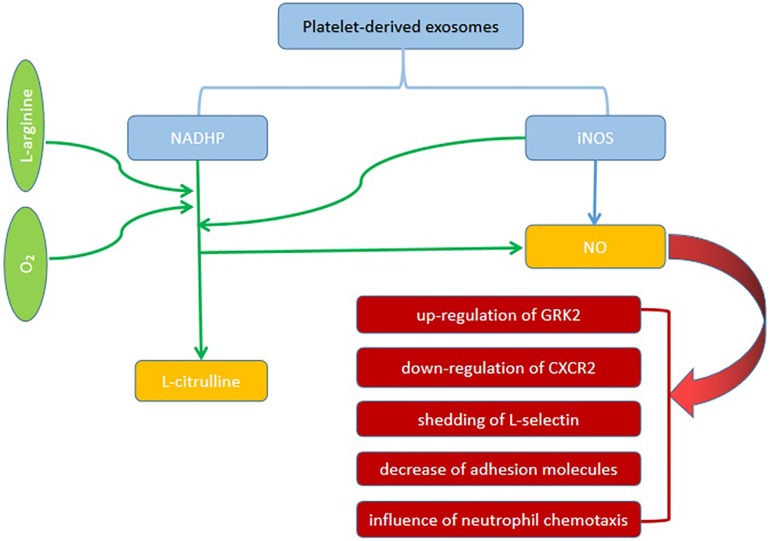
The roles of platelet-derived exosomes in SCM. Platelet-derived exosomes contain NADPH oxidase subunits similar to phagocytes can produce NO. Also, another component from exosomes, iNOS, can produce NO that is related to myocardial dysfunction in sepsis. NO can cause the up-regulation of GRK2, down-regulation of CXCR2, shedding of L-selectin, decrease in adhesion molecules, and influence of neutrophil chemotaxis.

Exosomes are small cell-derived vesicles originate from leukocytes, platelets and dendritic cells, etc. (Monteiro et al., 2017). Exosomes are a double-edged sword that can protect and cause SCM. The roles of exosomes in SCM is mainly considered from two mechanisms that are exosomal nicotinamide adenine dinucleotide phosphate (NADPH) and microRNA-223 (Monteiro et al., 2017). A study has shown that exosomes can induce vascular apoptosis and myocardial dysfunction by the mechanisms that are related to inflammation and oxidative stress (Real et al., 2018). In patients with sepsis, increased platelet-derived exosomes containing NADPH oxidase subunits similar to phagocytes in blood can help to produce ROS. Therefore, inhibiting the secretion of platelet exosomes would be beneficial for patients with sepsis. A study used GW4869 (a neutral sphingomyelinase inhibitor for blocking exosome generation) to investigate the effects of blockade of exosome release on the production of inflammatory cytokines and sepsis-induced myocardial dysfunction, suggesting that GW4869 deceased production of pro-inflammatory cytokines *in vitro* and inflammatory response *in vivo* via the inhibition of exosome generation. In addition, the attenuation of cardiac dysfunction and improvement of survival are noted in septic mice (Essandoh et al., 2015). Also, another component from exosomes, iNOS, can produce NO that is related to myocardial dysfunction in sepsis (Monteiro et al., 2017; Spiller et al., 2019). Figure 1 is the Illustration of the roles platelet-derived exosomes in SCM. On the contrary, MiRNAs are non-coding segments of RNA, which regulate the transcription of specific proteins. Studies have shown that miR-223 is down-regulated in patients who died of sepsis. miR-223 can inhibit the expression of endothelial cell adhesion molecule (ICAM-1) and negatively regulates transcription activator 3 (STAT3). Studies have shown that miR-223 found in exosomes and derived from mesenchymal stem cells (MSC) has a protective effect on cardiac function (Monteiro et al., 2017; Ge, 2019). Less amounts of miR-223 from MSC-derived exosomes are observed in blood in patients with septic shock, suggesting an impact of exosomes on cardiac dysfunction and mortality (Monteiro et al., 2017). Despite the inconsistencies regarding the role of exosomes in SCM, the association between SCM and exosomes has been established from current studies.

### Imbalance of Calcium Homeostasis in Cardiac Myocytes

In sepsis, decrease in density of L-type calcium channels and down-regulated sensitivity to calcium in myocardia myofilaments lead to decreased intracellular free calcium concentration and imbalance of calcium homeostasis, which eventually leads to decreased calcium-binding troponin and contractility (Dalton and Shahul, 2018). High mobility group box (HMGB) protein increases intracellular ROS level by interacting with TLR4, thereby increasing oxidative stress and phosphorylation of ryanodine receptor in cardiac myocytes. Meanwhile, increased ROS can enhance Ca^2+^-mediated Ca^2+^ leakage in SR, Ca^2+^ depletion from SR, and damage in myocardial excitation-contraction coupling (Kakihana et al., 2016). A study has shown beneficial effect of TLR4 inhibitor (TAK-242) through preventing SR Ca^2+^ leak in septic mice. Coincidentally, TLR4 deficiency significantly improved cardiac function and corrected abnormal Ca^2+^ handling in septic mice (Yang et al., 2018), which indicate that the critical role of TLR4-dependent SR Ca^2+^ leak in the development of SCM.

**FIGURE 2:**
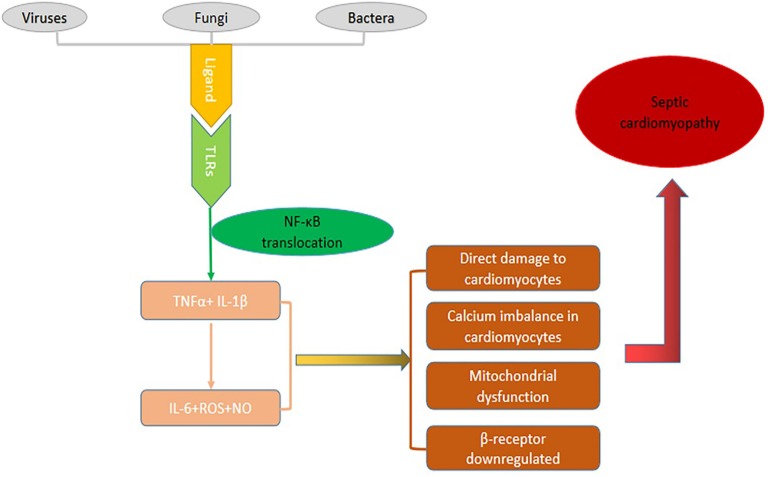
The pathogenic mechanism of SCM. Specific components called ligands of viruses, bacteria or fungi bind to TLRs then go through a series of cascade reactions that cause NF-κB to be transcribed into the nucleus, causing the expression of inflammatory factor genes and producing a large number of inflammatory mediators. These inflammatory factors can cause a series of direct damage to cardiovascular dysfunction, disequilibrium of calcium homeostasis, mitochondrial dysfunction, down regulated expression of β adrenaline receptor, and eventually lead to cardiac dysfunction.

The pathogenesis of SCM is extremely complex and our manuscript tends to discuss various mechanisms involved in SCM. Current studies indicate that the occurrence of SCM is the result of multiple factors including superantigen interaction with TLRs, then increase expressions of TNF-α and IL-1β that stimulate the immunocytes to produce other pro-inflammatory factors such as IL-6 as well as ROS. A large number of inflammatory cytokines and ROS can cause a series of direct damage to cardiovascular dysfunction, disequilibrium of calcium homeostasis, mitochondrial dysfunction, down regulated expression of β adrenaline receptor, and eventually lead to cardiac dysfunction (Figure 2).

## Diagnosis of Septic Cardiomyopathy

Currently, there is no unified international definition for SCM. In the 1980s, SCM was defined as the decrease in left ventricular ejection fraction (LVEF) and ventricular dilatation during sepsis (Beesley et al., 2018). However, LVEF depends profoundly on loading conditions, it has been increasingly acknowledged to be an inaccurate marker of intrinsic cardiac function largely. Now, some groups define SCM as an acute cardiac dysfunction syndrome caused by sepsis, which has nothing to do with ischemia (Beesley et al., 2018). In addition, it has one or more of the following characteristics: (i) decreased ventricular contractility; (ii) left ventricular dilation under normal or low filling pressure; (iii) right ventricular dysfunction and/or left ventricular dysfunction with a reduced response to fluid infusion (Martin et al., 2019). Currently, the challenges of defining SCM include: (i) how to evaluate cardiac function under the condition of high preload and postload; (ii) how to obtain longitudinal echocardiography data about cardiac function throughout the pathogenetic process (Sanfilippo et al., 2019).

To date, there is no gold diagnostic standard for SCM. Most clinical studies use LVEF < 50% as a diagnostic criteria (Ruian and Chen, 2018). Based on previous studies, diagnostic criteria of SCM should include two aspects: the presence of myocardial systolic and/or diastolic disorders, and the exclusion of other conditions leading to cardiac dysfunction. Here, with the exception of LVEF, we will discuss some other markers that may contribute to the diagnosis of SCM.

### Indicators Used to Assess Cardiac Function

Echocardiogram: Echocardiography and doppler imaging can detect abnormalities in systolic and diastolic function of the heart in patients with severe sepsis or septic shock. There are three main indicators, namely myocardial performance index (MPI), mitral ring systolic displacement (MAPSE) and global longitudinal strain (GLS). MPI, also known as Tei index, is a parameter measured by echocardiography (Beesley et al., 2018). The formula for MPI is: MPI = (ventricular isometric systolic time + ventricular isometric diastolic time)/ejection time. Some studies have indicated that left ventricular MPI is positively correlated with BNP and cardiac Troponin I (cTnI), and negatively correlated with LVEF. In a study of 47 patients with sepsis, Nizamuddin et al. (2017) found that deterioration of MPI within 24 h of admission was associated with increased 90-day mortality. MAPSE is an indicator used to assess global and local systolic function of the left ventricle. A small sample prospective study by Havaldar (2018) showed that MAPSE could predict mortality from SCM. Recently, some studies on speckle tracking echocardiography (STE) are performed, which can monitor GLS that refers to the changes in the length of myocardium during the period of contraction and the end of diastole (Jiahui and Cai, 2018). A large amount of data shows that GLS is more helpful for the early recognition and diagnosis of SCM than LVEF reduction (defined as left ventricular systolic dysfunction) (Jiahui and Cai, 2018). Ehrman et al. (2019) proposed that GLS was the preferred method for studying the relationship between left ventricular systolic function and prognosis in the patients with SCM. However, no diagnostic criteria of GLS is available for SCM currently. Vasques-Novoa et al. (2019) also reported that myocardial edema detected by magnetic resonance imaging in three patients with SCM. Magnetic resonance may be a promising modality for the diagnosis of SCM. Although auxiliary instrumental examination is not specific for SCM, it is an adjunct to early detection of SCM in patients with sepsis.

### Modified Shock Index (MSI) and Some Other Biological Markers

Modified shock index (MSI) refers to heart rate/mean arterial pressure, which can reflect both systolic and diastolic functions. Jayaprakash et al. (2018) conducted a retrospective study on 624 patients with severe sepsis or septic shock and concluded that the increase of MSI in the early stage (12 h) was correlated to the incidence of cardiac dysfunction, sequential organ failure assessment (SOFA) score and mortality. MSI was found to be promising predictors in febrile patients with sepsis. However, no single cut-off values of MSI was found to have an optimal accuracy for prediction of sepsis-related outcomes like SCM. Further studies are required to assess the incorporation of MSI in a multi-item scaling system for the prediction of SCM.

Some biological markers, such as B-type natriuretic peptide (BNP) and troponin I are elevated in patients with SCM, which have no diagnostic value specifically (Ruian and Chen, 2018). Chen et al. (2019b) analyzed the gene expressions from patients with SCM and proposed that CCL2, STAT3, MYC, and Serpine1 might be potential biomarkers or therapeutic targets for SCM. Although there is no conclusive evidence that these markers are associated with SCM, they may provide some clues for diagnosing SCM.

For the diagnosis of SCM, cardiac dysfunction caused by other diseases should be excluded. It is important to distinguish it from acute coronary syndrome. Coronary angiography and coronary computed tomography angiography (CCTA) can determine whether there is myocardial ischemia. But it is a risky examination for the patients with sepsis. Some studies have suggested using ultrasonic myocardial perfusion technology to examine myocardial perfusion abnormalities in the area of impaired myocardial wall in patients with sepsis. SCM and coronary syndromes can be distinguished by this method, but it is still invasive (Ruian and Chen, 2018). Also, other cardiomyopathies should be excluded, such as hypertrophic cardiomyopathy, dilated cardiomyopathy. These diseases usually have a long history and are slower to be developed.

## Treatment

Due to the uncertainty of pathogenesis of SCM, no effective disease-modifying treatment is currently available. The strategies to deal with SCM is to control the primary disease to prevent the occurrence of secondary SCM. Specific treatment for SCM is still being developed. Currently some drugs and device-based therapy have been used clinically.

### Clinical Treatment

#### Drug Therapy: Levosimendan, Natriuretic Peptide, Combination of Chinese and Western Medicine

Levosimendan is a calcium ion sensitizer, which can directly bind to troponin to increase myocardial contractility, but there is no significant change in heart rate and myocardial oxygen consumption. Studies have shown that levosimendan can effectively reduce the plasma lactic acid in sepsis and increase the myocardial contractility, but its application cannot reduce usage of norepinephrine and has no significant improvement in the mortality of SCM (Chang et al., 2018).

Natriuretic peptide, a recombinant human brain natriuretic peptide (rhBNP), can bind to human endogenous natriuretic peptide receptor. Natriuretic peptide not only can expand the arteries and veins, urinate and expel sodium to reduce the preload and afterload of the heart, but also can inhibit renin-angiotensin-aldosterone system (RAAS) and sympathetic nervous system, which has been widely used clinically for treating heart failure via effectively relieving hemodynamic abnormalities and cardiac dysfunction. Shi et al. (2015) reported that a patient with SCM caused by intestinal obstruction and diffuse peritonitis was recovered using natriuretic peptide. Yin et al. (2016) conducted a prospective study to observe 42 patients with sepsis complicated with cardiac dysfunction, showing that natriuretic peptide significantly improved the heart rate, mean arterial pressure, BNP, acute physiology and chronic health evaluation (APACHE-II) score, peripheral vascular resistance index (SVRI) and extracellular pulmonary fluid index (ELWI).

Combination of Chinese and western medicine: Clinical studies have shown the application of anti-infection and organ support therapy (MOST) accompanied by traditional Chinese medicine (fu zheng jie du hua yu granules) can further reduce acute physiology and chronic health evaluation (APACHE-II) score and other indicators such as procalcitonin (PCT), BNP and cTnI, in patients with SCM (Haiyun et al., 2016). Animal experiments have also shown that tanshinone IIA, a member of the major lipophilic components extracted from the root of Salvia miltiorrhiza Bunge, has a protective effect on myocardial injury in septic rat model (Dekun et al., 2016). However, further investigations are needed to clarify its mechanism of beneficial effect in SCM.

#### Non-drug Therapy: ECMO, IABP, Blood Purification

##### ECMO

In septic patients complicated with SCM, extracorporeal membrane oxygenation (ECMO) is a feasible rescue strategy. Some approaches have been reported that application of anti-infection combined with ECMO has been used to treat the patient with SCM and septic shock caused by infection and hemorrhage of ileal diverticulum (Liu et al., 2017). Vogel et al. (2018) conducted retrospective analysis and concluded that the survival rate in patients with SCM could reach up to 75% after 4 days of veno-arterial-venous (VAV) ECMO treatment.

##### IABP

Intra-aortic balloon counter-pulsation (IABP) supports cardiac function by unloading contractions and enhancing relaxation. The former can lower systolic blood pressure and the latter can raise diastolic blood pressure. A retrospective study was performed among 38 cases with the application of IABP, suggesting that IABP is effective for the patients with cardiac shock by increasing mean arterial pressure and reducing dosage of catecholamine during the acute phase of sepsis. However, no improvement in long-term survival was observed (Takahashi et al., 2019). Kenshiro Wada also reported that a patient combined with chronic cardiac insufficiency was recovered using V-A ECMO and IABP (Wada et al., 2019).

Blood purification has long been used to treat sepsis. Recently, Andreja Sinkovic reported that a patient with lymphoma, splenectomy and autologous bone marrow transplant and receiving chemotherapy, and accompanying severe pneumococcal infections, septic shock, SCM and unacceptable drug therapy, was subjected to blood purification. The reductions in the level of IL-6, lactic acid deposition and the dosage of vascular vasopressors, improvement of left ventricular systolic function and clinical features were observed after treatment for 36 h (Sinkovic et al., 2018). CytoSorb is a non-temperature, sterile disposable endotoxin and cytokine sorbent that reduces circulating cytokines such as IL-1β, TNF-α, IL-6, etc. (Gruda et al., 2018; Ankawi et al., 2019). A retrospective study by Brouwer et al. (2019) showed that continuous renal replacement therapy (CRRT) combined with Cytosorb improved the 28-day survival rate for septic shock compared with CRRT alone. All those methods have been applied clinically. However, only a few cases have been reported. A large-scale randomized clinical trial and prospective study are needed to better understand and evaluate the value of these approaches to treat SCM.

### Potential Treatments

#### Gene Therapy

Some studies suggested that the pathogenesis of sepsis is the damage of micro-vessels caused by “Genomic Storm”(Hawiger, 2018). Bacteria, fungi, infectious agents and viral nucleic acids bind to TLRs, causing the activation of stress response transcription factors (SRTFs), such as NF-κB, and activated protein 1 (AP-1), which in turn activate multiple genes encoding pro-inflammatory cytokines and chemokines, leading to septic shock and multiple organ dysfunction (Hawiger, 2018). Based on this observation, gene therapy for sepsis and SCM has been proposed. Studies have demonstrated that miR-21-3p inhibitors can improve cardiac dysfunction and mitochondrial ultrastructure damage caused by LPS, suggesting that miR-21-3p may be a potential target for SCM treatment (Wang et al., 2016). Zheng et al. (2017) proposed that miR-135a may serve as a therapeutic target in SCM because miR-135a can aggravate sepsis-induced inflammation and myocardial dysfunction possible via activation of p38 MAPK/NF-κB pathway. An et al. (2018) provided evidence that miR-146a can decrease pro-inflammatory cytokines and suppress apoptosis via inhibition of NF-κB activity by targeting TRAF6 and IRAK1. Cao et al. (2019) reported that miR-23b prevent NF-κB activation via inhibiting TRAF6 and IκκB, resulting in significant alleviation in cell injury induced by LPS as well as improvement in cell survival rate. We believe that gene therapy is the preferred method for SCM therapy in the future because it is targeted therapy with low side effects.

#### Mitochondrial Targeted Therapy

Structural and functional disorders of mitochondria affect the production of energy in cardiac myocytes, resulting in cardiac dysfunction. Maintaining the stability of mitochondrial structure and protecting its function have become the target of treating septic myocardium. Melatonin can restore the physiological functions of mitochondria and endoplasmic reticulum, maintain the stability of cytoskeleton, and thus improve cardiac function in septic mice (Zhang et al., 2019). The study on molecular mechanism has shown that melatonin attenuates the expression of BAP31 that interacts with mitochondria-localized proteins and regulates mitochondrial function (Zhang et al., 2019; Zhong et al., 2019). Kokkinaki et al. (2019) showed that chemically synthesized diglucoside (LGM2605) can reduce the accumulation of cardiac ROS, protect mitochondrial function in heart, reverse myocardial injury, and improve the survival rate in a mouse model of sepsis.

#### Inhibition of Inflammatory Mediators

Independent growth factor I (GFI-I) can inhibit the expression of NF-κB and TNF-α, thus inhibiting LPS-induced inflammatory response and apoptosis of HPC2 cells (Zheng et al., 2017). 3,3′-Diindolylmethane (DIM) is a potential therapeutic drug with scavenging free radicals and antioxidant effects. Studies have shown that DIM can significantly inhibit the expression of IL-6 and TNF-α induced by LPS, suggesting that DIM may be a new perspective for treating SCM (Luo et al., 2018). Qiangxin 1 formula effectively inhibited the expression of IL-1β, TNF-α, thus protecting the cardiac function of sepsis (Xu et al., 2018). Tan’s team demonstrated that hydrogen gas (H2) had a protective effect on cardiac insufficiency in LPS-induced sepsis in mice by blocking nuclear translocation of NF-κB (Tan S. et al., 2019). Supplementation of exogenous brain-derived neurotrophic factor (BDNF) can increase the level of BDNF in cardiac myocytes, improve cardiac dysfunction, reduce oxidative stress, and increase the survival rate in septic animals. Honda et al. found that the remote ischemic conditioner (RIC) could improve the ventricular function, cardiac output and survival rate in an LPS-induced septic mouse model (Honda et al., 2019). RIC may be a useful tool to improve the cardiomyopathy induced by sepsis clinically. Based on these observations, to reduce the level of inflammatory factors and regulate inflammatory signal are still the key for the treatment of SCM.

The treatments discussed in this section are only from animal experiments and have not been applied clinically. Whether or not the genetic response in animal models can mimic human inflammatory disease is controversial.

## Conclusion

Septic cardiomyopathy, although it is reversible at early stage, has a high mortality rate because its pathogenesis is not well-understood. In terms of diagnosis and treatment, it is an important subject in clinical and basic research. Previous studies in this area have been limited by poor diagnostic strategies that only relied on LVEF reduction. In this review, we have not only discussed pathogenesis of SCM in detail, but also introduced some other approaches that are associated with the diagnosis of SCM. Early detection and intervention of SCM in patients with sepsis can reduce mortality. For example, MSI is considered as a “predictor” of SCM (Jayaprakash et al., 2018), and MAPSE can predict mortality in SCM (Havaldar, 2018). A large number of new studies are needed to improve the understanding pathogenesis of SCM. It is believed that in the near future, the pathogenesis of SCM can be clarified and specific targeted therapeutic drugs can be developed to reduce the mortality of SCM.

## Author Contributions

HL and WW conceived and wrote the manuscript. ML, QM, and HR revised the manuscript. All authors listed wrote the manuscript and approved for publication.

## Conflict of Interest

The authors declare that the research was conducted in the absence of any commercial or financial relationships that could be construed as a potential conflict of interest.
